# Modifying platelets at their birth: anti-thrombotic therapy without haemorrhage

**DOI:** 10.3389/fphar.2024.1343896

**Published:** 2024-03-18

**Authors:** Conor Feely, Nitika Kaushal, Pier Paolo D’Avino, John Martin

**Affiliations:** ^1^ Centre for Clinical Pharmacology, Institute of Health Informatics, University College London, London, United Kingdom; ^2^ Department of Pathology, University of Cambridge, Cambridge, United Kingdom; ^3^ Division of Medicine, University College London, London, United Kingdom

**Keywords:** megakaryocyte, platelet, thrombosis, therapeutics, ploidy

## Abstract

Cardiovascular disease is a leading cause of death. The current approach to the prevention of arterial thrombosis in cardiovascular disease is dependent on the use of therapies which inhibit the activation of platelets. Predictably these are associated with an increased risk of haemorrhage which causes significant morbidity. The thrombotic potential of an activated platelet is modifiable; being determined before thrombopoiesis. Increased megakaryocyte ploidy is associated with larger and more active platelets carrying an increased risk of thrombosis. The reduction in the ploidy of megakaryocytes is therefore a novel area of therapeutic interest for reducing thrombosis. We propose a new therapeutic approach for the prevention and treatment of thrombosis by targeting the reduction in ploidy of megakaryocytes. We examine the role of a receptor mediated event causing megakaryocytes to increase ploidy, the potential for targeting the molecular mechanisms underpinning megakaryocyte endomitosis and the existence of two separate regulatory pathways to maintain haemostasis by altering the thrombotic potential of platelets as targets for novel therapeutic approaches producing haemostatically competent platelets which are not prothrombotic.

## 1 Introduction

Cardiovascular diseases are the leading cause of death globally; 85% of all deaths being due to ischaemic heart disease (IHD) or non-haemorrhagic stroke (Héctor, 2018). Cardiovascular diseases pose a huge personal, social, and economic burden across global healthcare. Despite addressing risk factors and their management, this disease remains one of the world’s biggest health challenges. The current treatment and prevention strategies for ischaemic heart disease and cerebrovascular disease rely mainly on antiplatelet therapy to prevent arterial thrombosis. These drugs are flawed as they universally demonstrate an increase in bleeding risk. Understanding the evolutionary biology of megakaryocytes and platelets, and the control mechanisms that regulate megakaryocyte (MK)-platelet interaction will be important in generating new therapeutic approaches. Here we address the more fundamental question of the mammalian thrombotic phenotype and how we might modify it without inhibiting the haemostatic potential of circulating platelets. We propose that new therapeutic ideas are needed to escape from the therapeutic link between antithrombosis and haemorrhage. Pursuing more specific ways of modulating platelets while not increasing the risk of bleeding may produce therapeutics superior to current therapeutic strategies. We aim to stimulate thinking which may lead to a conceptual leap in research aimed at identifying more efficient and safer antithrombotic therapies.

## 2 The current therapeutic paradigm

### 2.1 Current therapy

Antiplatelet medications are central to the current paradigm for the prevention of a range of arterial thrombotic events including myocardial infarction, stroke, and peripheral vascular disease (Héctor, 2018). Because the primary role of platelets is to limit haemostasis, the side effect of bleeding is the key obstacle to thrombus prevention via platelet inhibition (Baigent et al., 2002). The TIMI bleeding criteria (see Table 1) originally used in trials investigating thrombolysis for myocardial infarction, defines three groups of bleeding as major, minor, or minimal (Bovill et al., 1991). Variations of these criteria persist in modern dual antiplatelet therapy (DAPT) trials. The definition of “minor bleeding” includes clinically overt bleeding with up to a 4.9 g/dL drop in haemoglobin. These definitions may underplay the risk that these drugs pose to patients, e.g., minimal bleeding includes a drop of up to 2.9 g/dL in haemoglobin, possibly giving a false sense of security. The severity of blood loss associated with these therapies is significant and it is concerning that decades of drug development in antiplatelet agents have, in many cases, led to increasing rates of bleeding. Accepting these definitions of bleeding in clinical trials underplays the true risk of this treatment and suggests that the present standard treatment is more dangerous than the bleeding classification nomenclature suggests.

**TABLE 1:** TIMI bleeding criteria.

**TIMI Bleeding Criteria (3):**
1. Major
• Any intracranial bleeding (excluding microhaemorrhages <10 mm evident only on gradient-echo MRI)
• Clinically overt signs of haemorrhage associated with a drop in haemoglobin of ≥5 g/dL or a ≥15% absolute decrease in haematocrit
• Fatal bleeding (bleeding that directly results in death within 7 days)
2. Minor
• Clinically overt (including seen on imaging), resulting in haemoglobin drop of 3 to <5 g/dL or ≥10% decrease in haematocrit
• No observed blood loss: ≥4 g/dL decrease in the haemoglobin concentration or ≥12% decrease in haematocrit
• Any overt sign of haemorrhage that meets one of the following criteria and does not meet criteria for a major or minor bleeding event, as defined above:
◦ Requiring intervention (medical practitioner-guided medical or surgical treatment to stop or treat bleeding, including temporarily or permanently discontinuing or changing the dose of a medication or study drug)
◦ Leading to or prolonging hospitalization
◦ Prompting evaluation (leading to an unscheduled visit to a healthcare professional and diagnostic testing, either laboratory or imaging)
3. Minimal
• Any overt bleeding event that does not meet the criteria above
• Any clinically overt sign of haemorrhage (including seen on imaging) associated with a <3 g/dL decrease in haemoglobin concentration or <9% decrease in haematocrit
**Comment:**
The criteria used to assess bleeding need re-evaluation. “Minor bleeding” includes a drop in haemoglobin that is clinically significant and of major concern to patients and clinicians. The terms “minor” and “minimal” are misleading in this context and undermine the gravity of haemorrhagic side effects as a major limitation of this treatment

Aspirin is a well-established antiplatelet therapy in cardiovascular disease (Sneader, 2000). It acts by irreversibly blocking the cyclooxygenase (COX) activity of prostaglandin synthase 1 and 2 (COX-1 and COX-2), the initial enzymes in the prostaglandin synthesis pathway. Currently, the European Society of Cardiology (ESC) does not recommend the use of aspirin for primary prevention of myocardial infarction due to the increased risk of bleeding (Lang, 2018). However, the American College of Chest Physicians (ACCP) suggest using aspirin for primary prevention of myocardial infarction in some diabetic patients (Glenn et al., 2016). The discrepancy in the advice from the ESC and the ACCP indicates the difficulty of balancing the risk of bleeding against the antithrombotic effect of aspirin. The underlying mechanism of aspirin with blockade of both thromboxane and prostacyclin is the cause of the dilemma. The disparity in recommendations also reflects the difficulty arising from the complexity of platelet biology and the difficulty in achieving the correct balance of risk using the current antiplatelet regime. Additionally, the ASPREE trial of 19,114 older adults demonstrated that aspirin monotherapy in the elderly results in a significantly higher rate of major haemorrhage and did not achieve its primary clinical indication of reducing cardiovascular disease versus placebo (McNeil et al., 2018).

The addition of a P2Y12 receptor antagonist to COX-1 inhibition aimed to achieve a stronger platelet inhibition and reduce thrombotic events. Trials exploring the efficacy of DAPT (dual antiplatelet therapy) regimens looked at the primary end points of non-fatal MI, non-fatal stroke, and mortality due a cardiovascular cause (See Table 2 (Yusuf et al., 2001; Montalescot et al., 2009; Wallentin et al., 2009)). DAPT consisting of aspirin and a P2Y12 inhibitor is now widely recommended for the medical management of acute coronary syndrome (Glenn et al., 2016; Lang, 2018). The strategy of pursuing dual platelet blockade via different mechanisms predictably leads to an increased risk of haemorrhage. De-escalation and modification of DAPT in clinical practice demonstrates the pressing difficulty in managing the bleeding risk profile against anti-thrombotic effect. DAPT is routinely de-escalated to a single antiplatelet agent at 12 months. Choice of P2Y12 agent is tailored to a patient’s bleeding risk and relative risk of thrombosis, demonstrating that the bleeding profile of these drugs often results in a compromise of anti-thrombotic potential.

**TABLE 2:** Example trials in the history of anti-thrombotic drug development.

Study	Population	Additional treatment	Control group	Research group	Primary efficacy end point	Efficacy results	Primary safety end point	Safety results
CURE (2001) Yusuf et al. (2001)	Adults with ACS without ST elevation	Aspirin	Placebo	Clopidogrel	Composite of death from cardiovascular causes, nonfatal myocardial infarction, or stroke	Clopidogrel: 9.3%	CURE bleeding criteria for major haemorrhage	Clopidogrel
						Placebo:		3.7%
						11.4%		Placebo
						(Relative risk with		2.7%
						clopidogrel as compared with placebo, 0.80; 95 percent		(Relative risk, 1.38; 95 percent confidence interval,
						confidence interval, 0.72 to 0.90; *p* < 0.001)		1.13 to 1.67; *p* = 0.001)
Triton-TIMI 38 (2007) Montalescot et al. (2009)	Patients with ACS with or without ST Elevation Scheduled for PCI	Aspirin	Clopidogrel	Prasugrel	Composite of the rate of death from cardiovascular causes, nonfatal myocardial infarction, or nonfatal stroke	Clopidogrel: 12.1%	TIMI bleeding criteria: Major bleeding	Clopidogrel: 1.8%
						Prasugrel: 9.9%		Prasugrel: 2.4%
PLATO (2009) Wallentin et al. (2009)	Adults with ACS – with or without ST elevation	Aspirin	Clopidogrel	Ticagrelor	Composite of death from vascular cause, stroke or MI at 12 months	Clopidogrel:	PLATO bleeding criteria: Major bleeding	Clopidogrel:
						11.7%		11.2%
						Ticagrelor:		Ticagrelor:
						9.8%		11.6%
						hazard ratio, 0.84;		*p* = 0.43
						95% confidence interval [CI], 0.77 to 0.92; *p* < 0.001		

Optimising thrombosis prevention at the expense of increasing major haemorrhage is the defining obstacle of the current treatment paradigm with these agents. There is research into finding alternative independently acting agents with dual antiplatelet activity. Notable examples include GPIIb/IIIa antagonists and PAR inhibitors.

Thrombin activates platelets via the protease activated receptors (PARs). Vorapaxar is an oral PAR1 inhibitor. However, trial data shows that its addition to aspirin and clopidogrel does not lead to significant reduction in thrombotic events but leads to increased haemorrhage (Tricoci et al., 2012). These results indicate that increased blockade of platelet activation, in this case COX-1 inhibition, ADP receptor antagonist and PAR inhibition, reaches a threshold where thrombosis prevention does not improve but haemorrhagic consequences disproportionately increase.

Many platelet adhesion molecules have been identified and have been considered for potential therapeutic targets, including integrins and the immunoglobulin superfamily (Xu et al., 2016). This provides alternative approach to preventing thrombosis by stopping the development of fibrin and fibrinogen attachments between platelets led to the discovery of GPIIB/IIIA blockers. There are three commonly used agents: the monoclonal antibody (Abciximab) and the small molecule inhibitors (tirofiban and eptifibatide). The earlier agents were administered intravenously only and were largely used in a peri-interventional setting. Recommendations on their use include the caveat of careful consideration of the associated bleeding risk and paucity of evidence for benefit in addition to DAPT (Siddiqui T and Dikshit, 2013; Glenn et al., 2016; Lang, 2018). GPVI is exclusively expressed on platelets and MKs (Dütting et al., 2012) It is a platelet collagen receptor involved in platelet activation and the subsequent actions of adhesion, degranulation, and potentiation of the prothrombotic signal targeting a receptor central to multiple aspects of acute thrombosis that is present only on the target cells was an attractive target for a novel agent (Xu et al., 2016). However, platelet plug formation can still occur in the presence of GPVI dysfunction as demonstrated in cases of autoimmune antibody formation against GPVI and congenital abnormalities of the protein which can cause a mild bleeding phenotype (Takahashi and Moroi, 2001; Dütting et al., 2012). Pharmacological inhibition of GPVI has been attempted via multiple methods including monoclonal antibodies, soluble humanised Fc fusion protein (revacept), and humanised antigen-binding fragment (glenzocimab) (Renaud et al., 2020). Glenzocimab demonstrates some promising early phase clinical trial data in a placebo-controlled trial with the primary end points assessing safety, mortality and intracranial haemorrhage (Mazighi et al., 2024). The glenzocimab arm showed lower rates of intracranial haemorrhage and all-cause mortality. Efficacy data for this novel approach is awaited from ongoing and future clinical trials.

### 2.2 A flawed strategy

Current treatment and prevention strategies for ischaemic heart disease and cerebrovascular disease rely mainly on antiplatelet therapy to prevent arterial thrombosis. However, this treatment falls short of ideal in several respects including its failure to prevent thrombosis in a high proportion of patients (9.8% in the PLATO trial) and its risk of serious haemorrhage. Attempted progress within the field of antiplatelet therapy has demonstrated optimisation of various drug characteristics but ultimately repeats the same barrier to progress: haemorrhage. The reliance on iterative research into antiplatelet combinations and the pursuit of ever more powerful platelet inhibitors appears to be a flawed strategy.

## 3 Understanding the megakaryocyte-platelet axis

In this section we will review the uniqueness of mammalian MK and platelet biology. We hope to stimulate research with new ideas derived from an understanding of the evolution-derived role the platelet has in preventing bleeding. We propose that the therapeutic target of modulating platelet production may produce agents that prevent thrombosis without increasing the risk of bleeding (Patrono et al., 2004).

### 3.1 Normal megakaryocyte-platelet behaviour

Platelets are small anucleate fragments of cytoplasm with no genomic DNA. They contain MK derived mRNA and the requisite machinery to undertake protein synthesis to a limited degree. Their structure and function are therefore determined, to a large extent, by the MK mRNA and thus intrinsic variation in behaviour in physiology or disease will be determined by the MK. MKs are large platelet-progenitor cells, average volume 4,700fl, which originate in the bone marrow and exhibit the rare characteristic among mammalian cells of being highly polyploid (Harker and Finch, 1969; Mazzi et al., 2018). MKs replicate chromosomal DNA but fail to complete cell division in a process called endomitosis and can contain up to 128N DNA content (normal diploid cells have a 2N content of DNA).

Normally, the MK-platelet system is in dynamic equilibrium, typically producing 1 × 10^11^ platelets per 24 h in humans (Trowbridge and Harley, 1984). Each MK can produce between 2000 and 3000 platelets and platelets on average survive for 7–10 days in the circulation (COHEN and LEEKSMA, 1956; Ratnoff, 1987). Notably, in acute blood loss, the initial response is the creation of larger platelets and not an increase in the number of platelets in the circulation (Ratnoff, 1987). These larger platelets are denser than platelets of equivalent size not responding to platelet loss, they contain more mitochondria per unit volume, more receptors per plasma membrane surface area, and are more responsive to agonists of aggregation compared to controls (Kaushansky, 2015). If the dynamic equilibrium of platelet production is disturbed by platelet loss, it returns to normal steady state after 30 days in man.

Thrombopoietin has a major role in megakaryopoiesis, acting on all bone marrow progenitors of MKs (Broudy et al., 1995). Furthermore, *in vitro* and *in vivo* models demonstrate that thrombopoietin increases MK ploidy by increasing endomitoses, with thrombopoietin-deficient mice models showing changes in small and low ploidy MKs (Gurney et al., 1994). Although the discovery of this ligand gives insight into MK-platelet maturation, the wider regulatory mechanism of thrombopoiesis in haemostasis is still only partly understood. The regulation of platelet production in creating a pro-haemostatic phenotype in response to bleeding is likely also to be involved in a causal pro-thrombotic phenotype in cardiovascular disease (Brown et al., 1997).

Lower order vertebrates (i.e., birds and reptiles) have a nucleated cell equivalent to the MK called the thrombocyte (Ratnoff, 1987). The thrombocyte originates in the bird or reptile bone marrow and then circulates in the peripheral blood performing both immunological and haemostatic roles (Ferdous and Scott, 2015). These nucleated cells aggregate to achieve haemostasis in a manner equivalent to aggregation of platelets. The biological uniqueness of the platelet plus its universality among mammals suggests a single event in evolution gave rise to platelets about 220 million years ago (Martin and Wagner, 2019). The fragmentation of MKs to platelets gave a powerful haemostatic evolutionary advantage because of the small size of the platelet, very large surface area and quick granule release. Platelets are a highly evolved and effective mechanism of haemostasis that probably allowed the development of invasive placentation in mammals (Martin and Wagner, 2019). This theory suggests that, because neonatal mammals depend on maternal lactation, survival of the mother after birth confers a survival advantage. However, the origin of platelets indicates a novel and powerful cellular system under novel control. Understanding of the control mechanism, since it must be involved in the fundamental mechanism of mammalian haemostasis, should give rise to novel and potentially powerful means of preventing thrombosis related to platelet behaviour.

Furthermore, the cell volume distribution curve of platelets is unique. In cells that undergo mitosis, cell volume distribution is Gaussian, owing to the size of the two daughter cells being approximately the size of the mother cell. However, platelet volume distribution exhibits the biologically unique distribution of being log-Gaussian (Martin et al., 1983a). This is consistent with a process of physical fragmentation which suggested the theory of platelet production in the pulmonary circulation. Initially hypothesised as an effect of platelet ageing but evidence thereafter suggests that platelets do not change in size or density as they age but rather that platelet heterogeneity is determined at thrombopoiesis (Martin et al., 1983a). The environment and physiological circumstances in which platelet production occurs may also influence platelet reactivity. Thus, if platelet heterogeneity is determined at thrombopoiesis, then alteration in size, density and reactivity will be preceded by changes in MKs (Martin et al., 2012).

It was observed that mature MKs lie adjacent to sinusoids in the bone marrow and create cytoplasmic protrusions called proplatelets. One hypothesis suggests that these then fragment into platelets from bone marrow sinusoids to the blood stream (Kaushansky, 2015; Lefrançais et al., 2017). These conclusions were drawn primarily from *in vitro* experiments. However, there is growing evidence in support of an alternative hypothesis which suggests that MKs leave the bone marrow and fragment into platelets in the pulmonary circulation; also, it has been suggested that the mechanisms of proplatelet formation might occur in the pulmonary circulation as part of the process of MK pulmonary fragmentation (Lefrançais et al., 2017; Ouzegdouh et al., 2018).

Furthermore, it is important to appreciate that MKs behave differently *in vivo* and *in vitro* which may explain the history of conflicting evidence on this topic (Bornert et al., 2021). The pulmonary vasculature as a site of platelet release may offer a site for the delivery of novel antithrombotic therapeutic agents via the lungs. Importantly, it is possible that proplatelets may be produced during the process of pulmonary fragmentation, thus unifying the two hypotheses.

There is evidence that the regulatory mechanisms of platelet production from MKs, which confer an evolutionary advantage via enhanced haemostasis may, in post-reproductive life, confer a potential disadvantage, if activated inappropriately, in the form of a prothrombotic phenotype (Martin et al., 2012). Platelet density and volume directly reflect the composition of the MK cytoplasm from which they come. Not only are they an indicator of platelet reactivity but also, indirectly, reflect MK reactivity. Mean platelet volume (MPV) (which correlates with platelet density) has been the most common quantitative variable measured across studies as a predictor of myocardial infarction, as density is technically more exacting to assess (Martin et al., 1983a). Thrombopoiesis in physiological equilibrium produces an inverse relationship between MPV and platelet count in both man and other mammalian species (Martin et al., 1983a; von Behrens, 1972). In times of platelet destruction, this equilibrium is changed so that in the short-term, individual platelets are produced which each have greater haemostatic potential, (under the control of a rapid response signal). A chronic activation of this rapid response signal in combination with the production of more prothrombotic MKs persistently would produce a chronic prothrombotic state. As shown in Ratnoff (1987) the whole platelet production/destruction equilibrium is reset over time following acute destruction. Some individuals, at risk of arterial occlusion, with chronic low grade platelet destruction, e.g., on hypofunctional endothelium, the system may be set in a prothrombotic mode (Martin et al., 2012).

Animal models demonstrate that the kinetics of platelet production, with an increased mean size after injection of an anti-platelet serum, is similar to the changes seen in humans following a decreased platelet count associated with platelet loss in cardiopulmonary bypass (Martin et al., 1982; Martin et al., 2012).

New research from Petzold et al. (2022), explores the role of neutrophils in thrombopoiesis which gives vital insight into the interplay between sterile inflammation and platelet production. In this work, neutrophils are seen to “pluck” proplatelet extensions of the MK, demonstrating a mechanosignalling component to the production of platelets. This is of particular interest when modelling the pathophysiology of coronary artery disease resulting in thrombosis. It allows us to visualise the direct impact of sterile inflammation present in damaged endothelium on the production of increased numbers of immature, prothrombotic platelets.

Furthermore, MK volume and ploidy have a linear relationship with, and increase in response to, a reduced circulating platelet mass (Martin et al., 1982; Martin et al., 1983b; Martin et al., 2012). This is relevant as evidence suggests MKs with higher ploidy are associated with larger and more reactive platelets in steady state production as well as states of increased platelet destruction (Martin et al., 1983b). Reactive platelets may play a central role in the pathogenesis of several diseases, especially atherosclerosis and atherothrombosis (as discussed above).

### 3.2 Pathological megakaryocyte-platelet behaviour in vascular disease

There is evidence that an association exists between Megakaryocyte-platelet pathology and ischaemic heart disease (IHD), cerebrovascular disease and ischaemia of the leg. Studies assessing patients with atherosclerotic disease, showed an increase in MK ploidy and a corresponding increase in platelet mass over normal controls (Corash, 1989; Brown et al., 1997). MK size (which correlates with ploidy) increased in patients who suffered a recent MI compared to controls and in men suffering from sudden cardiac death compared to age matched controls who suffered traumatic deaths (Trowbridge et al., 1984). Similarly, men suffering from acute myocardial infarction (AMI) had a higher MPV (within 12 h) compared to controls (Martin et al., 1983c; Trowbridge and Martin, 1987). Evidence (see below) suggests the increase in MK ploidy and MPV precedes ischaemic events and has a causal role (Klovaite et al., 2011). In a rabbit model, platelet destruction led to the development of large high ploidy MKs, associated with a significant increase in atherosclerotic lesions of the aorta (KRISTENSEN et al., 1990a). Platelets arising from these MKs probably have a causative role in both the pathogenesis of atheroma formation and the result of ischaemic events as these are highly reactive dense platelets.

The bleeding time in patients with AMI following a single 300 mg dose of aspirin was shortened compared to controls; suggesting the presence of more active platelets in AMI patients (Kristensen et al., 1990b). This may suggest greater cyclo-oxygenase activity in patients with MI and as platelets have no nuclei, this enzyme must have been produced in the MKs prior to platelet release into the circulation. Such platelets also have a higher expression of plasma membrane glycoprotein IIb/IIIa compared to healthy controls (Giles et al., 1994).

The evidence reviewed so far notes the changes in platelets after AMI. It may be argued these platelet changes occur as a result of a pathological change rather than being the cause of It. However, as the lifespan of circulating platelets is about 10 days, at least 90% of the platelets in which MPV was measured were circulating before the AMI, suggesting, that MPV is increased before complete occlusion of the coronary artery. In MI, the whole of the platelet volume distribution curve is shifted to the right, to higher values, suggesting that the changes in MPV volume occurred at thrombopoiesis in the MK (Trowbridge and Martin, 1987). One possibility is that the increase in platelet volume is secondary to increase in platelet turnover (either acute or chronic) caused by platelet consumption on the altered endothelium preceding the thrombotic event. This consumption would trigger the fast control system discussed above. However, the fast signal may be applied slowly if platelet destruction is slow, e.g., from slow platelet consumption on hypofunctional endothelium. Chronic platelet consumption would gradually signal gradual change in MK ploidy gradually increasing thrombotic risk. The gradual platelet consumption would be caused by gradual decrease in endothelial function caused by chronic exposure to thrombotic risks such as hypertension, smoking, diabetes, and lack of exercise (which has an effect on endothelial mechano receptors). All these would decrease the production of nitric oxide and prostacyclin from endothelial cells, increasing platelet consumption. If the effect of the risk factors is chronic then the increase in platelet volume would be chronic and slow. Thus, in this situation the raised MPV may be seen as a causal link between systemic endothelial cell change and coronary occlusion by hyperactive platelets. Furthermore, increased MK size in the bone marrow of men who died from sudden cardiac death compared to controls is evidence that high ploidy MKs were present before the fatal cardiac event (Trowbridge et al., 1984).

Platelet behaviour (as reflected in platelet volume and density) after MI has predicted outcome. Martin et al. (1991) studied 1716 men in whom MPV was elevated 6 months after AMI; it was a powerful predictive risk factor for both recurrent AMI and death at 2 years follow up. MPV did not correlate with other known risk factors such as blood pressure, cholesterol or smoking and denser larger platelets appeared to be an independent risk factor for repeat AMI. This study further links MPV as an independent etiological factor in AMI as well as supporting a primary causative role. These findings were supported by other studies which examined the association between MPV and clinical outcomes in patients with non-ST-segment elevation AMI (Aksu et al., 2009; Azab et al., 2011; Taglieri et al., 2011). In patients undergoing percutaneous coronary intervention (PCI), baseline MPV was a predictor of coronary restenosis (Goncalves et al., 2011). The Copenhagen General Population study of 39,531 men and women was important in showing a causal link between MPV and cardiovascular disease in the general population over 10 years (Klovaite et al., 2011). The results demonstrated that MPV was the strongest, and an independent, risk factor in predicting the risk of MI compared to other known risk factors (Klovaite et al., 2011). Therefore, there is a body of evidence that platelets are causal in the thrombus formation that occludes the coronary artery causing myocardial infarction. Modulation of platelet production in patients at risk of cardiovascular disease is a novel therapeutic target. We propose the long-term reduction in MK ploidy as a means to reduce thrombotic risk as either primary or secondary prevention.

## 4 Three novel therapeutic strategies

Identifying novel therapeutic targets for the treatment and prevention of thrombotic events would be an important advance. A more specific way of modulating the prothrombic phenotype to reduce risk of thrombosis while not increasing the risk of bleeding would be superior to current therapeutic strategies. The following areas are suggested as research themes in this area that we believe could become a new branch of therapeutics.

### 4.1 Modulating the receptor mediated events

Increase in mean platelet volume (and density) indicating increased platelet reactivity are powerful independent risk factors for myocardial infarction and stroke. The increase in MK ploidy and mean platelet volume following loss or destruction of platelets occurs rapidly after acute platelet destruction as demonstrated in an animal model and in man (Martin et al., 1983b; Trowbridge et al., 1984). Both in animals and man the occurrence of large prothrombotic platelets occurs within 24 h. The speed of change indicates a mechanism that is a receptor mediated event with a ligand that could be a therapeutic target. The rapid change in volume of platelets suggests that the initial response comes from mature MKs as there would not be enough time for new MKs to mature to produce platelets in response within 24 h. The anucleate platelet is not capable of undergoing this change itself.

This indicates a feedback pathway that involves a sensor of platelet destruction or consumption and a factor that is released via the sensor which is a ligand for a receptor on MKs which are mature enough to produce platelets. The site and nature of the receptor is now unknown. However, the question is scientifically tractable by employing the same methodology used in the discovery of c-kit ligand, where a receptor was discovered prior to the ligand; initially known as c-kit ligand and later thrombopoietin (Yarden et al., 1987). The same receptor may mediate the effects of slow platelet consumption by damaged endothelium which would mimic chronic haemorrhage and therefore activate a feedback mechanism designed to maintain haemostasis in haemorrhage. It is postulated that this mechanism would be activated in later life, (after its evolutionary purpose of maintaining haemostasis at mammalian birth becomes redundant), in the context of atherosclerosis and endothelial damage. Development of an antagonist of the receptor mediating the effect of the ligand involved would therefore oppose the increase in platelet size and density induced by the vascular pathology. The ligand will be a protein which would be amenable to drug development. Medicinal chemistry and structural biology could identify active peptides from the ligand which could be refined into peptoids. These peptoids would be the basis of small molecules antagonists. The therapeutic objective of this process would be an orally available molecule with a plasma half-life that allowed it to be used as a chronic prophylactic treatment in patients at risk of thrombosis. Another strategy would be to identify the signal transduction pathway of the receptor and develop inhibitors of the enzymes involved. Importantly an antagonist of the slow response would inhibit the production of prothrombotic platelets but would not affect the production of haemostatically competent platelets. Therefore, this strategy would be antithrombotic without being pro haemorrhagic. This principle is central to the ideas in this review: a novel therapeutic objective of resetting the physiology of platelet production.

### 4.2 Genes controlling polyploidy and potential targets

The success of any precisely designed therapy relies on its ability to target the unique aspects of the pathology, as this can help to both increase efficacy and reduce toxicity. One unique aspect of MK biology is that the cells can become highly polyploidy and importantly, as described in Section 3.1, increase in MKs ploidy directly correlates with the presence of large prothrombotic platelets and risk of myocardial infarct. Therefore, one possible strategy for reducing the number of large prothrombotic platelets, while producing haemostatically competent platelets, could be to interfere with the mechanisms that control MK polyploidization.

MK polyploidization occurs during their differentiation from precursors known as promegakaryoblasts (or MK precursors), which initially proliferate through normal mitotic divisions and begin to produce factors important for platelet function. However, at one point during the differentiation process, promegakaryoblasts become highly polyploid through a series of aborted cell divisions, or endomitoses, to produce megakaryoblasts. After polyploidization ends, the megakaryoblasts finally mature into MKs by increasing their cytoplasm through enhanced protein synthesis and by producing specific secretory granules and a demarcation membrane system (DMS) (Kennedy and Lowe, 2022). MK differentiation and polyploidization appear to be controlled by the same signalling pathways and part of a co-ordinated genetic program, but unfortunately the origin and function of this polyploidization have yet to be fully elucidated (Yarden et al., 1987; Mazzi et al., 2018).

It was initially debated whether MK polyploidization resulted from failure in chromosome segregation (karyokinesis) or in the separation of the two daughter cells at the end of mitosis (cytokinesis). However, various studies strongly indicate that cytokinesis failure is likely the main origin of polyploidy in MKs (Geddis et al., 2007; Lordier et al., 2008). This includes the observation of multinucleate cells (which is the expected outcome of cytokinesis failure) in MKs derived from cord blood or differentiated *in vitro* from human pluripotent stem cells (Moreau et al., 2016). However, the presence of polylobate nuclei rather that multiple nuclei in mature, highly polyploid MKs indicates that a combination of both karyokinesis and cytokinesis defects is the most likely cause of polyploidy in these cells (Lordier et al., 2012; Mazzi et al., 2018). Together, this evidence suggests that cytokinesis failure could initially cause the formation of tetraploid (4N) megakaryoblasts, which could then increase their ploidy through subsequent endomitoses characterized by a combination of chromosome segregation and cytokinesis defects that are both typically observed in polyploid cells.

Studies aimed at understanding in detail the molecular mechanisms underlying the process of endomitosis responsible for polyploidization could lead to the identification of molecules and pathways that could be targeted to interfere with the polyploidization process and in turn reduce the formation of large prothrombotic platelets. As increase in MK ploidy is achieved through repeated cycles of endomitosis, the alterations to the process of cell division responsible for polyploidization must be transmitted from the mother cell to the daughters through an inheritable, most likely epigenetic, mechanism. Therefore, we recently suggested that a possible unbiased experimental approach to identify such alterations could be a comparative multi-omics analysis of the epigenome, transcriptome, and proteome of megakaryoblasts of different ploidy status differentiated *in vitro* (Martin and Paolo D Avino, 2022). As the vast majority of cell division processes are regulated by reversible post translational modifications (PTMs), such as phosphorylation and ubiquitylation, it is reasonable to assume that these multi-omics studies could reveal alterations in the function and/or activity of one or more PTM enzymes as the underlying cause of MK polyploidization (Wieser and Pines, 2015; Cuijpers and Vertegaal, 2018). Small molecule inhibitors of several mitotic PTM enzymes, mostly being developed for cancer therapies, are already available and could therefore represent potential “off-the-shelf” anti-thrombotic drugs operating via this mechanism. These drugs could be tested in phase II trials in patients at high risk of vascular disease (Jiang and Zawacka-Pankau, 2020; Kennedy and Lowe, 2022).

One interesting and potentially exploitable aspect of MK polyploidization is that these cells must be able to bypass or silence the surveillance mechanisms that prevent polyploid cells re-entering the cell cycle (Ganem and Pellman, 2007). A key player in limiting proliferation of polyploid cells is the stress-responsive transcription factor p53, which acts as the “guardian of the genome” and is mutated in about 50% of cancers (Kennedy and Lowe, 2022). Therefore, it is likely that p53 must be silenced and/or repressed in order for megakaryoblasts to go through multiple rounds of endomitoses. If the multi-omics studies would confirm this, then another possible therapeutic approach to prevent MK polyploidization could be to target the E3 ubiquitin ligase MDM2 responsible for p53 degradation. This strategy, already currently considered for cancer therapies could increase p53 levels thereby limiting MK polyploidization, returning the ploidy distribution to a non-thrombotic profile (Jiang and Zawacka-Pankau, 2020; Koo et al., 2022).

There is increasing evidence of sub-populations of MKs both anatomically and functionally (Sun et al., 2021; Asquith et al., 2024). The latter insight developed from single cell analysis of ploidy describe specialised subpopulations of MKs that can function in an immune capacity. This is of particular interest to our proposal on how to approach novel therapeutic avenues through manipulation of ploidy. If a subpopulation of MKs primarily responsible for the increased production of large more thrombogenic platelets can be identified then this will offer a more specific therapeutic target.

When conceptualising a novel agent consideration should be given to the relatively low frequency of MKs as the target cell, particularly if we aim to target a subpopulation. Multiple pools of MKs exist in the body (lung, spleen, bone marrow) but even at the most populous site, the bone marrow, there are usually only 2-4 per high powered field of the bone marrow biopsy (Asquith et al., 2024).

Targeting rare cells requires targeted therapies to minimise off-target effects. There are several novel drug designs that could be employed such as antibody-drug conjugates or antibody-protein conjugates (Tsuchikama et al., 2024) However, a targeted oral small molecule inhibitor modulating MK precursors may be a simpler and equally efficacious approach.

### 4.3 Fast and slow control mechanisms

Novel research on the transcriptional characterisation of human MK polyploidisation and lineage commitment demonstrates that two distinct transcriptional states exist for low and high ploidy MKs (Choudry et al., 2021). The transcriptional landscape of a 32N MK and a 4N MK are markedly different. The 32N MK displays upregulated genes localising to the cell surface membrane, cytoplasm, internal membrane, mitochondria, nucleus, and secreted components. Analysis of the difference in transcriptional patterns between the high and low ploidy states suggests that lower ploidy MKs produce proteins related to platelet function and cell surface proteins. However, the transcriptional focus of higher ploidy MKs appears to be on the localisation of proteins into alpha granules and dense granules among other roles. Secretion of these granules from the platelet at aggregation is important in thrombus formation. Single cell analysis of bone marrow MKs from patients undergoing coronary artery bypass graft operations for acute myocardial infarction comparing matched controls demonstrated upregulation of genes in MKs related to thrombus formation such as PPBP and the AMPA glutamate receptor, GRIA1 (Choudry et al., 2021). The identification of genes upregulated with increasing ploidy, both supports the hypothesis that MK changes are causal in thrombogenesis and aids our understanding of potential therapeutic targets. Research focussed on identifying the transcriptional signatures of pathological thrombopoiesis could transform therapeutics in this field.

Furthermore, the journey from haematopoietic stem cell to high or low ploidy MKs appears to be more complex than previously understood. In addition to lineage commitment to become a MK, subpopulations of HSCs were identified which then became either high ploidy or low ploidy MKs. Suggesting there is a yet unknown regulatory mechanism to influence platelet phenotype which affects the haematopoietic stem cell and subsequent stages of maturation. However, this work did support the hypothesis that a dual control system for the production of MKs and therefore platelets, exists (Choudry et al., 2021).

## 5 Conclusion

Cardiovascular disease is a major cause of mortality internationally. Our current therapeutic model relies heavily on the use of therapeutics that inhibit circulating platelet activation. The current practice involves the use of dual antiplatelet therapy following an acute myocardial infarction which includes aspirin and a P2y12 receptor antagonist. This approach does not completely prevent the recurrence of thrombosis in the form of a second myocardial infarction at 12 months and haemorrhagic side effects are a significant cause of morbidity. Inhibition of platelet activation is a blunt approach to a complex process and refining this approach should reduce unintended side effects such as haemorrhage. By modifying their ploidy distribution MKs directly influence the size and activity of platelets. There is evidence for the role of a feedback loop in haemostasis that increases the prothrombotic potential of the platelet in response to haemorrhage. We suggest that this appropriate evolutionary mechanism becomes pathological in cardiovascular disease and the prothrombotic state then created by high ploidy MKs directly contributes to the formation of acute thrombotic events such as myocardial infarction.

We propose a new paradigm for chemical and biological therapeutic research and development in the field of arterial thrombosis by exploiting the receptor mediated events that lead to the increase in ploidy of MKs, by exploiting the molecular mechanisms underpinning endomitosis and therefore polyploidy and finally by attempting to define fast and slow feedback mechanism targets in haemostasis and thrombosis that modify MK-platelet biology. This original therapeutic approach aims to reduce thrombotic risk while not increasing the risk of haemorrhage.
